# Supplementary material for: Complexome profiling of the Chlamydomonas psb28 mutant reveals TEF5 as an early PSII assembly factor
Source: Plant Cell. 2025 Mar 17;37(6):koaf055. doi: 10.1093/plcell/koaf055 (PMC12164590; doi:10.1093/plcell/koaf055)
Supplement: koaf055_Supplementary_Data [file koaf055_supplementary_data.zip › psb28_interactive.zip!/plots/intensity/qualityPlots/peptides/NUOP3_Cre02.g100200.t1.2.html]

### Cre02.g100200.t1.2

Trivial name: NUOP3  
SeqCoverage [median]: 18.80  
Mass: 22.32 kDa  
  
 

| Sequence | MissedCleavage | Mass [Da] | wtIntensity | muIntensity | Score |
| --- | --- | --- | --- | --- | --- |
| DIVQDYQR | 0 | 1035.50 | 23704.81 | 12879.09 | 131.04 |
| LVEVTVVDMNGIR | 0 | 1443.78 | 1995.81 | 3858.52 | 129.16 |
| VQNVDPESDDGLAVK | 0 | 1584.76 | 12463.26 | 7172.20 | 220.31 |
| VQNVDPESDDGLAVKR | 1 | 1740.86 | 30047.27 | 24351.15 | 148.57 |
|  |  |  | 68211.2 | 48261.0 |  |
